# Alcohol, neuronal plasticity, and mitochondrial trafficking

**DOI:** 10.1073/pnas.2208744119

**Published:** 2022-07-11

**Authors:** John Hernandez, Karla R. Kaun

**Affiliations:** ^a^Department of Neuroscience, Brown University, Providence, RI 02912

Consumption of alcohol creates a sense of euphoria, reduces inhibition, and increases sociability and impulsivity (1). The age at which alcohol is first experienced is a key factor contributing to the likelihood to misuse alcohol (2). However, the impacts of the first experience of alcohol on the molecules in the brain at these key developmental stages are not well understood. Knabbe et al. (3) endeavored to address the neuromolecular alterations resulting from acute alcohol by combining hippocampal proteomics with somatosensory and motor cortex protein, dendrite, axon, and mitochondrial analysis in adolescent mice. Evidence from this array of preparations led to the hypothesis that alcohol disrupted mitochondrial trafficking, and using *Drosophila* they demonstrated a functional role for mitochondrial trafficking in cue-induced alcohol preference.

The cross-assay and cross-species approach outlined in Knabbe et al. (3) proved to be an effective way of discovering how alcohol hijacks brain mechanisms. Animals from flies to humans maintain functionally consistent neurotransmitter systems, neural circuit mechanisms, and molecular pathways underlying reward (4). Flies and mice also demonstrate behavioral responses to the pharmacological properties of alcohol that look remarkably similar to those in humans, highlighting the conserved neurobiological basis of alcohol on the brain (5).

Although the similarity in brain structure and genetic sequences between mice and humans allows us to easily intuit the application of results in mice to humans, the observation that alcohol affected similar proteins across different brain regions speaks to the broad effects of alcohol on the brain (3). This also allowed the authors to take advantage of multiple assays to study mechanism across several levels of analysis to get a detailed understanding of the underlying cell biology (Fig. 1). Ultimately this led to the discovery of a role for mitochondrial trafficking in how alcohol affects neuronal activity, structural plasticity, and behavior (3).

**Fig. 1.**
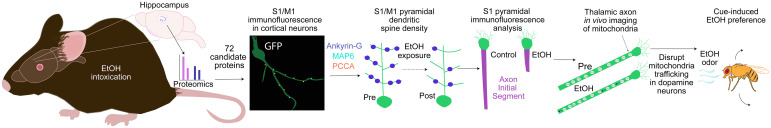
Cross-assay and cross-species approaches to understanding how alcohol affects the brain. Knabbe et al. (3) used proteomic approaches in postnatal-day-30 mice to examine the impacts of acute alcohol exposure at a critical developmental stage. Subsequently, candidate synaptic proteins were examined in the primary somatosensory (S1) and motor (M1) cortex. Alcohol exposure increased synaptic boutons loss, decreased axon initial segment length, and increased mitochondria motility. When mitochondrial trafficking in dopamine neurons is disrupted, adult *Drosophila* are unable to form appetitive alcohol memories.

The conservation in mechanism across several brain regions in mice also hinted that this role for alcohol may translate across species. Indeed, the authors demonstrated a functional role for mitochondrial trafficking in cue-induced alcohol preference in *Drosophila* by in vivo manipulation of mitochondria trafficking in specific cell types using sophisticated genetic tools (6) (Fig. 1). The remarkable convergence upon a single mechanism by leveraging several assays across brain regions and species demonstrates the effectiveness of interdisciplinary and collaborative work.

## Mitochondria and Alcohol Addiction

Mitochondria are the powerhouses of the cell. These membrane-bound cell organelles generate most of the chemical energy needed to power a cell’s biochemical reactions, and trafficking of mitochondria along axons is necessary for structural plasticity, membrane dynamics, and, ultimately, cell survival (7, 8). Localization of mitochondria at synaptic sites is critical for proper neuronal function (9). Mitochondria must be trafficked, docked, and maintained at synaptic sites where they not only provide energy but they also act as scaffolds for activity-dependent local translation of synaptic machinery (9, 10).

Given the importance of mitochondria in neuronal plasticity, and the profound effects of alcohol on neuroplasticity, it is perhaps not surprising that mitochondria play an integral role in alcohol-associated responses. Alcohol induces mitochondrial fragmentation that leads to myopathy in *Caenorhabditis elegans* similar to myopathy observed in humans abusing alcohol (11, 12). Similarly, alcohol reduces mitochondrial bioenergetics in the central nervous system in zebrafish (13). These effects appear to be conserved across species. Adolescent binge-like exposure reduces cognitive performance, hippocampal plasticity, and mitochondrial function in the rat hippocampus (14). Likewise, alcohol-induced hangover suppresses mitochondrial function in rats (15) and reduces mitochondrial-dependent motor performance in mice (16).

Knabbe et al. (3) built on this literature by first identifying synaptic proteins that were modulated by acute alcohol intoxication in the hippocampus of adolescent and adult mice, then demonstrating how alcohol reduced boutons in the cortex consistent with mitochondrial-dependent changes occurring at synapses in the hippocampus (Fig. 1). This led to the finding that mitochondrial trafficking was increased in cortical axons that derived from thalamic neurons up to 4 h after alcohol injection. To complement these data, an injection of alcohol suppressed performance on a go/no go task for at least 4 to 6 h, suggesting alcohol impacts reward-related behavior. As it is logistically difficult to demonstrate a causal role for mitochondrial trafficking in alcohol behavior in mice, the authors turned to *Drosophila* (Fig. 1). They demonstrated that when the protein complexes required for axonal mitochondrial transport (i.e., the Miro/milton/kinesin complex) in dopaminergic neurons were genetically disrupted, *Drosophila* were no longer able to make positive associations between an odor cue and the pharmacological properties of alcohol. Together, these interdisciplinary findings suggest that intoxicating doses of alcohol disrupt mitochondrial function and inversely that mitochondrial function is important for encoding appetitive alcohol memory.

## Mitochondria and Drug Addictions

Recent studies have broadly implicated mitochondria as targets through which drugs of abuse influence neuronal function and behavior. Neuronal cell culture studies demonstrate that cocaine (17), as well as amphetamine (18), exposure alters mitochondrial dynamics and can even induce apoptosis. Similary, methamphetamine has been identified to target mitochondria-related neuronal apoptosis in the hippocampus (19) and dopaminergic neurons in the substantia nigra (20). Synthetic opioids such as fentanyl alter mitochondrial structure in neuron cell culture (21). This affects general cell health since mitochondrial DNA copy numbers reflect the overall health of cells in opioid-dependent rodents (22). Even 3,4-methylenedioxy-methamphetamine (MDMA, also known as ecstasy) has been shown to induce mitochondrial swelling and outer membrane damage that underlies deficits in spatial memory formation (23). Together these data suggest that multiple abused psychoactive substances have impacts similar to alcohol on mitochondrial function in neurons, and consequently behavior.

## From Mechanism to Treatment

Alcohol broadly deteriorates human health when consistently consumed in high amounts. Alcohol consumption has been increasing in recent years, a trend which has been exacerbated by the COVID-19 pandemic (24, 25). Unfortunately, current treatment options only reach a small percentage of the population that needs them. Understanding more about how alcohol affects the brain is key for developing more effective and accessible treatments.

The real mystery is the myriad of ways through which alcohol can affect the brain. It can alter membrane channel permeability, acutely change neuronal excitability, chronically alter glial function, and both acutely and chronically alter chromatin structure, gene expression, and protein synthesis (26). So, which mechanism is the best one to pursue for developing effective treatments for alcohol use disorder? The acute effects on mitochondrial trafficking demonstrated in Knabbe et al. (3) suggest that one of the ways that alcohol can alter tissue health is through homeostatic mechanisms that occur as a result of alcohol’s influencing energy use in the cell. This could be a promising avenue for therapy due to the overlap with cancer, obesity, cardiovascular disease, neurodegeneration, and aging (27). The prominent comorbidity of alcohol use disorder with these diseases certainly suggests that this is an avenue worth pursuing.

## Change History

August 15, 2022: The text of this article has been updated; please see accompanying Correction for details.
